# Undescended Testicle (in the Horse)1Read before the Annual Meeting of the Ontario Veterinary Medical Association.

**Published:** 1896-02

**Authors:** S. Sisson


### UNDESCENDED TESTICLE (IN THE HORSE).[1]

BY S. SISSON, V.S.

THIS form of arrested development is frequently seen in our domestic animals, and, while interesting in all, is of special importance in the horse, as it usually interferes materially with his usefulness, and consequently greatly depreciates his value.

It is perhaps unnecessary to go into details as to the anatomical arrangement. Confining our attention to retentio abdominalis, what we find is briefly as follows:

The testicle is usually soft and imperfectly developed. It is enveloped by the peritoneum, which radiates from the testicle in three double folds. The anterior of these (representing the primitive mesorchism) contains the spermatic vessels and nerves. The posterior fold passes backward into the pelvic cavity and contains in its edge the vas deferens. The third one passes outward and downward into the inguinal canal (constituting the processus vaginalis), and contains the gubernaculum testis, presenting on its outer surface the imperfectly developed cremaster muscle. Such, briefly, is the arrangement of the peritoneum, which, of course, is identical with that found in the fœtus a short time before the descent of the testicle into the canal. Speculation as to the etiology of the condition would naturally be futile, but it may be remarked that the influence of heredity has frequently been observed.

Recognition of cryptorchism is usually easy, especially if the history of the case is available. The sexual appetite is often greatly exaggerated, constituting satyriasis, and coitus is usually vigorous but unproductive. Some cases become quite vicious, attacking other animals and even the attendant. In exceptional cases, where the animal is old or hard-worked, none of the preceding symptoms may be noticed, especially when he is kept from proximity to mares.

Frequently the head and crest are typically male, but this is far from being constant, notably where both testicles are retained, or where one has descended and been removed at the usual age. Further evidence is furnished by a careful ex-

1 Read before the Annual Meeting of the Ontario Veterinary Medical Association.

amination of the inguinal region revealing no scar on inspection nor the stump of the cord on manipulation. Sometimes the testicle can be detected per rectum, but if small and soft it is very elusive.

Two operative methods have been practiced, viz., removal through a flank incision or through the inguinal canal. The latter seems preferable in the horse, and is the one usually adopted. If carefully performed the perforation of the peritoneum is very small, very little air need pass into the cavity, and drainage can also be perfectly obtained.

The animal should be made to fast during twenty-four hours preceding operation, or better, a physic ball given two or three days previous, and in the interval a very limited allowance of food and water.

As a matter of course, all possible aseptic measures should be adopted as to hands, instruments, and the field of operation.

It is difficult to describe lucidly on paper methods of casting and securing, but it may suffice to state that the *sine qua non* is to avoid drawing the hind limbs forward. They should be well flexed (hip, stifle, and hock), and somewhat abducted. To secure the latter condition the spreader may be used, but is not essential.

The administration of chloroform is of very material assistance in operating. It abolishes the necessity for particular methods of securing and lessens the danger of prolapse of the intestine.

An incision through the skin and subcutaneous fascia is then made, either parallel to the median line, or, preferably, in an oblique direction, corresponding to the long diameter of the external inguinal ring. In either case it should begin about an inch from the median line and extend three or four inches. If careful the operator need not injure the subcutaneous abdominis artery and large satellite vein.

The external inguinal ring is then exposed by tearing asunder the loose sub-fascial connective tissue with both index fingers. The first and second fingers of the hand corresponding to the side being operated on are then introduced into the canal, where, of course, in many cases the testicle will be found. If not the operator will find instead the small processus vaginalis, which must not be mistaken for the degenerated spermatic cord and tunica vaginalis, and accepted as an indication of former castration. It may, however, be secured and used as a guide for the fingers in the canal, if unfamiliar with the operation.

Very little force is required in passing up the canal, and the fingers should be closely applied to Poupart's ligament until the point of perforation is reached. This should be made close to the internal inguinal ring and is best performed by a quick, unhesitating thrust with the index and middle fingers *during inspiration*, as Möller wisely advised.

Manipulation is then made with the two fingers over the surface which can be reached. If the testicle or epididymis is not encountered the fingers must be directed backward and search made for the vas deferens. This offers a characteristic cord-like sensation to the fingers, and its range of movement is quite limited, as it is retained in position by a peritoneal frænum about three inches in width at this part. If difficulty be experienced the assistance of the other hand in the rectum may expedite matters.

When the vas deferens is secured gentle traction on it brings the testicle up to the opening, through which it can be drawn usually without difficulty. It can then be removed in the usual manner, after which the wound may be cleansed, any blood removed, and a few stitches inserted if the tissues have not been bruised much.

It is only in *very* exceptional cases that it becomes necessary in operating to introduce the entire hand into the abdomen, and the routine practice of doing so cannot be too strongly condemned. Where the testicle cannot be drawn outside of the wound, owing to shortness of its peritoneal investment, the écraseur chain can be passed up sufficiently into the canal.

In horses new-growths of the undescended testicle are not so common as in the human subject and dog, but may of course be encountered. Cysts are much more common and, as in these cases, the cyst may be large (Möller records one containing ℥xvj of serum), and the testicle very small, they are somewhat troublesome. Degives punctures the cyst with the finger-nail, and then removal of the testicle is easy.

A few cases are recorded in which the testicle had contracted adhesions in the abdomen or pelvis. Such a condition fortunately seems very rare. It would necessitate introduction of hand (and possibly part of the arm), and separation of the adhesions. Naturally the prognosis would be grave.

